# COVID-19 nationwide lockdown and physical activity profiles among North-western Italian population using the International Physical Activity Questionnaire (IPAQ)

**DOI:** 10.1007/s11332-021-00745-8

**Published:** 2021-03-04

**Authors:** Federico Abate Daga, Samuel Agostino, Serenella Peretti, Luca Beratto

**Affiliations:** 1 Department of Medical Sciences, University of Turin, Turin, Italy; 2 Adapted Training and Performance Research Group, School of Exercise and Sport Sciences, University of Turin, Turin, Italy

**Keywords:** Health enhancing, Inactivity, Quarantine, Active lifestyle

## Abstract

**Background:**

The role of physical activity in enhancing health is well recognized. However, in the more industrialized countries, physical activity rates are declining, and the emerging COVID-19 pandemic may amplify this scenario. Thus, this study aimed to report the Italian population’s physical activity profile living in the North-western regions during the national lockdown.

**Methods:**

Physical activity was assessed using the official Italian short form of IPAQ, intended for Social Networking Services (SNS). Participation was voluntary, and people could fill the questionnaire simply by clicking on a specific link.

**Results:**

The final sample size was 612 Italians, male and female, equally distributed (49.1% male and 50.9% female). Data showed a percentage of 15.4% of inactive subjects (MET < 700), 61.8% of moderate-active (MET 700–2500), and 22.9% of active people (MET > 2500). Women were more inactive than men (*p* < 0.01; 57.4% vs 42.6%). Furthermore, participants were classified into four classes of age: 18–29; 30–44; 45–59; and 60–79. Class 18–29 was prevalent (*p* < 0.01, 68.3%) and showed higher levels of physical activity (*p* < 0.05).

**Conclusions:**

Italians living in North-western regions maintained a moderate amount of physical activity during the quarantine. This behavior should be encouraged, particularly among women that usually appear to be less active than men.

## Introduction

Physical inactivity is a significant risk factor for several chronic diseases, including cardiovascular diseases, diabetes mellitus, obesity, osteoporosis, and certain types of cancer [1–4]. Furthermore, lack of physical activity is also linked to premature mortality and shortening health span [5, 6]. Benefits of an active lifestyle are nowadays well recognized by the World Health Organization (WHO) [7] that promotes to reach a goal of 150 min/week of moderate-to-vigorous intensity physical activity such as walking, running, cycling, or swimming to prevent cardiovascular diseases and their comorbidities [8, 9]. However, in the major industrialized countries, physical activity rates seem to decline instead of increase [10], and the emerging COVID-19 pandemic amplified this scenario. The high potential spread of this virus [11] forced several countries to declare a nationwide lockdown. As a consequence, the possibility to practice physical activity has been severely reduced. On the opposite, moderate amounts of exercise improve immune system functions and reduce the risk of infections [12]. Thus, a certain amount of physical activity is significant to remain active and healthy, which might be very useful in fighting against COVID-19 [13]. Training at home can be a reasonable compromise to accomplish WHO recommendations of remaining fit and maintaining physical strength and endurance [7, 14–16]. Several studies have demonstrated the benefits of home training [17, 18], but they mainly investigated special populations as postmenopausal women, older adults with a high risk of bone fractures, and diabetes [17–20]. Therefore, information about the practice of home exercises among the whole population are still not available. Nevertheless, during the nationwide lockdown, home training represented the only chance to remain active. Consequently, monitoring physical activity levels during a nationwide lockdown might be interesting for public health. An excellent tool to investigate physical activity rate in a large population is the International Physical Activity Questionnaire (IPAQ). It was developed by various researchers from different countries, with support from the World Health Organization (WHO) and the US Centers for Disease Control and Prevention (CDC) [21, 22]. IPAQ’s unique feature is that it assesses all believed health-related physical activities that can take place in different settings. Thus, it is an appropriate tool to evaluate the home practice of physical activity. Therefore, the purpose of this study was to report the physical activity profile of the Italian population living in the North-western regions during COVID-19 national lockdown, using the short version of IPAQ [23] intended for use in Social Networking Services (SNS). The goal of the study intends to analyze physical activity habits according to age, gender and previous practice.

### Methods

### Study design and ethical considerations

The present exploratory study was conducted in Italy’s North-western regions during the quarantine due to the COVID-19 pandemic. Participants were asked to answer an online questionnaire and provided their informed consent to participation by virtually signing a statement. Participation was voluntary and anonymous, and the questionnaire was automatically interrupted in case of a negative response to the informed consent statement. The Institutional Research Ethics Board of the University of Turin approved this study (number of approvals: 251716).

### Participants

The survey was circulated via social network platforms (Facebook, Instagram, and WhatsApp) among adults 18 years or older residing in the Italian regions of Piemonte, Valle d'Aosta, and Liguria (North-western of Italy). Questionnaires submitted by people younger than 18 years of age or not living in the North-western of Italy were rejected. Only completed questionnaires have been considered valid for further analysis.

### Outcome measurements

The questionnaire was available online at the following link: (https://docs.google.com/forms/d/e/1FAIpQLSfQATecSqT2rX752Vy5WtT2b102RWxAF_7J7lxHu_i3nm48jQ/viewform?usp=sf_link.) between March 9 and April 10, 2020. This form was IP address case sensitive. Therefore, once completed, nobody could fill it again from the same IP address. First, we collected sociodemographic variables, such as gender, age, Body Mass Index (BMI), practiced sport (if any), and the related years of practice (Table 1). Successively, we submitted the International Physical Activity Questionnaire (IPAQ). The IPAQ questionnaire was developed to assess self-reported physical activity levels in adults aged 18–79 [21]. The IPAQ questionnaire collects information about the duration, frequency, and intensity of physical activity in four domains: (1) work, (2) transport, (3) domestic and gardening, and (4) leisure time during the last 7 days. This tool assessed low, moderate, and vigorous-intensity amounts (days/week, hours, and minutes per day) as well as the daily sitting time. From these values, we calculated the total number of physical activities per week and the total amount of moderate-to-vigorous physical exercise in terms of Metabolic equivalent tasks (MET) by multiplying durations, frequencies, and MET scores for each type of activity.

**Table 1:** Representation of sociodemographic variables. We collected gender, age, Body Mass Index (BMI), practiced sport (if any), and the related years of practice. BMI classes have been identified considering World Health Organization guidelines (WHO. Obesity and overweight. Fact sheet N°311. Geneva: World Health Organization, 2015)

Sex	Number	%	Age	Number	%	BMI	Number	%	Sport practice	Number	%	Years of practice	Number	%
Men	304	49.7	15–29	418	68.3	Underweight (< 18.5 kg/m^2^)	43	7.03	Yes	563	92%	0	79	12.91
Women	308	50.3	30–44	79	12.91	Normal weight (18.5 to 25 kg/m^2^)	478	78.1	No	49	8%	1–4	125	20.42
			45–59	85	13.89	Overweight 25 to (30 kg/m^2^)	85	13.89				5–8	109	17.81
			60–78	30	4.9	Obese I (> 30 kg/m^2^)	3	0.49				9 +	299	48.86
						Obesity II (30·0– < 35·0 kg/m^2^)	3	0.49						
						Obesity III (35·0– < 40·0 kg/m^2^)	0	0						

### Data analyses

Data were analyzed using SPSS, version 19.0 (SPSS Inc., Chicago, IL, USA), after being checked for outliers according to IPAQ guidelines (www.ipaq.ki.se). Frequencies and percentages have been calculated for each demographic characteristic and physical activity levels (walking, moderate or vigorous).

Chi-square statistics were calculated for both males and females by activity status. Physical activity and BMI were analyzed across age groups and years of sports practice using a One-way ANOVA with Bonferroni correction. Physical activity rate was calculated as follows: low activity (walking) = (3.3 × walking minutes x walking days); moderate activity = (4.0 × moderate activity minutes × moderate activity days); vigorous exercise = (8.0 × vigorous activity minutes × vigorous activity days. Furthermore, outcomes were classified into three categories: inactive (< 700 MET × week), moderately active (700–2500 MET × week), and active (> 2500 MET × week), according to the scoring system provided by IPAQ [21].

### Results

Eight hundred and twelve subjects filled the questionnaire. However, 200 answers have been rejected because participants did not meet the inclusion criteria. The remaining 612 (49.1% male and 50.9% female) answers were considered acceptable for further analysis.

Participants’ gender was equally distributed between male (49.1%) and female (50.9%), and BMI did not show any significant differences (males: 23.09 ± 2.5 kg*m^−2^; females: 21.37 ± 2.8 kg*m^−2^, *p* = 0.280). Furthermore, BMI data indicated that 78.1% of the screened population was within a normal range (22.23 ± 2.8 kg*m^−2^) while 13.9% (BMI 26.58 ± 1.2 kg*m^−2^) overweight. Furthermore, 92% of the participants declared regular physical activity (or sport) before lockdown, while only 8% maintained a sedentary lifestyle.

**Fig. 1:**
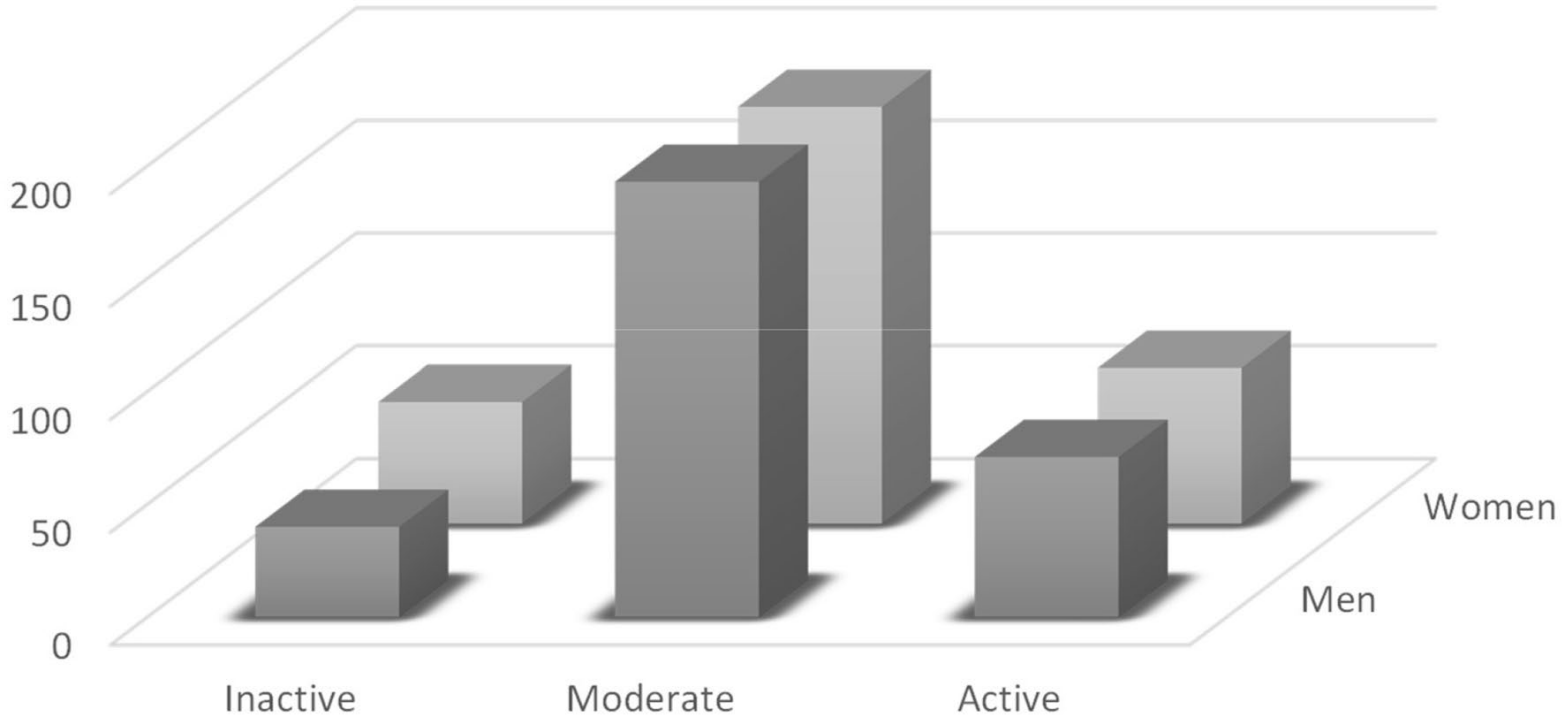
The proportion of North-western Italian population who are inactive, moderate-active, or active during lockdown according to gender. Moderate-active people are significantly prevalent (*p* < 0.01). No differences were registered between gender in all classes of physical activity profile

**Fig. 2:**
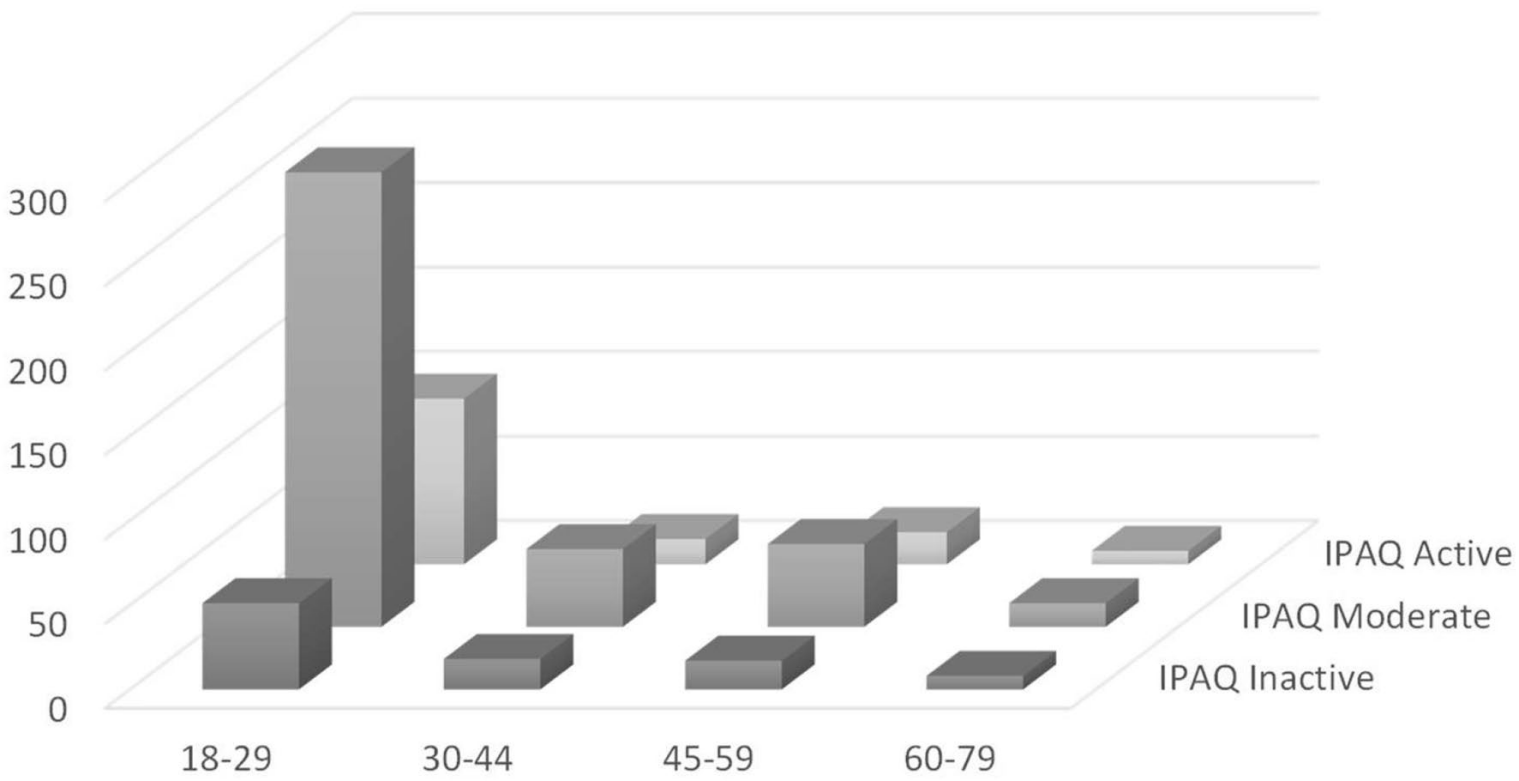
The proportion of North-western Italian population, moderate-active, or active during lockdown according to years of age. Class of age 18–29 is significantly prevalent (*p* < 0.05). Moderate-active people are prevalent (*p* < 0.01) in all classes of age

IPAQ outcomes are shown in Table 2. 15.4% of the participants were classified as inactive subjects (MET < 700), 61.8% of moderate-active (MET 700–2500), and 22.9% active people (MET > 2500) (Figs. 1, 2). Male and female did no show significant differences in MET (males: 50.1%, female: 49.9%; *p* = 0.59). A significant difference in MET values was observed considering sport practice years (< 1 year vs > 4 years; *p* < 0.001).

**Table 2:** Physical activity profile of the Italians leaving in North-western regions during lockdown. Moderate-active people were prevalent (*p* < 0.01) in both gender

Gender		IPAQ		
		Inactive	Moderate	Active
		94	378	140
Men	304	40	193	71
Women	308	54	185	69

Age ranks are represented in Table 3. Participants were classified into four classes of age: 18–29; 30–44; 45–59; and 60–79. People belonging to the class of age 18–29 were the most representative and showed higher levels of physical activity (68.3%, *p* < 0,05). No differences were identified within classes of age 30–44, 45–59, and 60–79 (*p* = 0.98). Women showed a prevalence among inactive subjects (57.4% vs 42.6%; *p* < 0.01), while no significant differences between sexes were identified between moderate-active and active participants (moderate: male 51.1%, female 49.9%, *p* = 0.997; active: 50.7 males, 49.3% females; *p* = 0.404) (Table 3).

**Table 3:** Physical activity profile of the Italians leaving in North-western regions during lockdown according to their years of age

Age	Number	IPAQ		
		Inactive	Moderate	Active
		94	378	140
18–29	418	51	269	98
30–44	79	18	46	15
45–59	85	17	49	19
60–79	30	8	14	8

Finally, males spent more time in walking activity than females (43.20% vs 38.60%; *p* < 0.01).

## Discussion

The present study aimed to investigate physical activity profiles during a nationwide lockdown using the IPAQ questionnaire submitted online through email or Social Networking Services (SNS). IPAQ was massively used to obtain data on health-related physical activity due to its versatility in submission and ease in data acquisition [24–26]. First, this study showed that just over half of the population reached by this survey resulted moderately active during lockdown (61.8%), independently from sex. Thus, people were moderate-active for at least 30 min or more a day for 5 days a week. The inactive population was 15.4%, while the active or very active part was 22.9%. These outcomes were fascinating, considering that they were acquired during the lockdown. Even if people were forced to stay at home, moderate physical activity was practiced anyway. Previous studies conducted in pre-COVID-19 emergency [24, 25] showed that in “normal life,” only nearly half of the population was moderate-active with an incoming alarming trend to a sedentary lifestyle [10]. On the opposite, this study showed that 61.8% of interweaved people were moderate-active, despite being locked at home. This fact could be described considering the most representative age of the participants. Range 18–29 was the largest with 68.3%, and tendentially young people are more active than adults or elderly [27]. Furthermore, the investigation was supposed to ask people if they practiced physical activity regularly before lockdown and how many years of experience they had in their training program. Outcomes showed that 92% of the participants practiced regular physical conditioning before the lockdown with at least 1 year of discipline experience. The more experienced (4 years of experience or more) were also the more engaged in-home training. Thus, this fact probably contributed to keeping high physical activity levels, even in an extraordinary situation such as a nationwide lockdown. It is well demonstrated that physical activity produces many benefits on the body and mind [28, 29]. Therefore, those who usually practiced physical training before lockdown may adopt alternative strategies to continue training themselves at home.

Another relevant outcome was the percentage of inactive or poor active people (less than 750 MET a week). In this survey, 15.4% of the participants declared to be inactive or weak active. A study of Hallal and colleagues [27] based on data from 122 countries reports that nearly a third of the adult population (31.1%) reveals a lack of physical activity. Even if still high, data of this survey underline that physical inactivity during the lockdown in the North-western Italian population was firmly under the average global levels. About this, we might hypothesize that recommendations from WHO and relevant scientists [13, 30] positively influenced people’s behavior.

Furthermore, females were more inactive than males (inactive female: 57.4% vs. 42.6% of inactive male). These results agree with other studies in which it is demonstrated as women are less active than men since childhood [31, 32]. This behavior belongs to psychosocial and cultural variables[33, 34] that lead women to be less engaged in physical activity programs. The outcomes of this study seemed to confirm this trend during a lockdown.

Finally, this study presents the following limitations. First, this report used the short form of IPAQ intended for Social Networking Services (SNS) and email. Thus, as is the case with any questionnaire, the respondents could have received more than one link or have experienced internet connection problems. Second, subjects were randomly reached through social networks or public or private internet websites that spontaneously sponsored this survey. However, Social Networking sites are problematic for elders to use because of computer illiteracy, lack of knowledge of Web 2.0 concepts, and format, navigation, and layout issues [35]. Thus, this fact could be the reason for a higher prevalence of young responders.

## Conclusion

As shown in the present study, people in Italy’s North-western regions conserved a moderate-active status despite the lockdown. This behavior was very interesting considering the running emergency. It should be encouraged to increase the number of physically active people, particularly among women who usually appear to be less active than men.
